# Comparing the Psychological Effects of Manikin-Based and Augmented Reality–Based Simulation Training: Within-Subjects Crossover Study

**DOI:** 10.2196/36447

**Published:** 2022-08-01

**Authors:** Shannon Toohey, Alisa Wray, John Hunter, Ian Waldrop, Soheil Saadat, Megan Boysen-Osborn, Gabriel Sudario, Jonathan Smart, Warren Wiechmann, Sarah D Pressman

**Affiliations:** 1 Department of Emergency Medicine University of California, Irvine Orange, CA United States; 2 Department of Psychological Science University of California, Irvine Irvine, CA United States

**Keywords:** augmented reality, AR, salivary cortisol, galvanic skin conductance, medical simulation, medical education

## Abstract

**Background:**

Patient simulators are an increasingly important part of medical training. They have been shown to be effective in teaching procedural skills, medical knowledge, and clinical decision-making. Recently, virtual and augmented reality simulators are being produced, but there is no research on whether these more realistic experiences cause problematic and greater stress responses as compared to standard manikin simulators.

**Objective:**

The purpose of this research is to examine the psychological and physiological effects of augmented reality (AR) in medical simulation training as compared to traditional manikin simulations.

**Methods:**

A within-subjects experimental design was used to assess the responses of medical students (N=89) as they completed simulated (using either manikin or AR) pediatric resuscitations. Baseline measures of psychological well-being, salivary cortisol, and galvanic skin response (GSR) were taken before the simulations began. Continuous GSR assessments throughout and after the simulations were captured along with follow-up measures of emotion and cortisol. Participants also wrote freely about their experience with each simulation, and narratives were coded for emotional word use.

**Results:**

Of the total 86 medical students who participated, 37 (43%) were male and 49 (57%) were female, with a mean age of 25.2 (SD 2.09, range 22-30) years and 24.7 (SD 2.08, range 23-36) years, respectively. GSR was higher in the manikin group adjusted for day, sex, and medications taken by the participants (AR-manikin: –0.11, 95% CI –0.18 to –0.03; *P*=.009). The difference in negative affect between simulation types was not statistically significant (AR-manikin: 0.41, 95% CI –0.72 to 1.53; *P*=.48). There was no statistically significant difference between simulation types in self-reported stress (AR-manikin: 0.53, 95% CI –2.35 to 3.42; *P*=.71) or simulation stress (AR-manikin: –2.17, 95% CI –6.94 to 2.59; *P*=.37). The difference in percentage of positive emotion words used to describe the experience was not statistically significant between simulation types, which were adjusted for day of experiment, sex of the participants, and total number of words used (AR-manikin: –4.0, 95% CI –0.91 to 0.10; *P*=.12). There was no statistically significant difference between simulation types in terms of the percentage of negative emotion words used to describe the experience (AR-manikin: –0.33, 95% CI –1.12 to 0.46; *P*=.41), simulation sickness (AR-manikin: 0.17, 95% CI –0.29 to 0.62; *P*=.47), or salivary cortisol (AR-manikin: 0.04, 95% CI –0.05 to 0.13; *P*=.41). Finally, preexisting levels of posttraumatic stress disorder, perceived stress, and reported depression were not tied to physiological responses to AR.

**Conclusions:**

AR simulators elicited similar stress responses to currently used manikin-based simulators, and we did not find any evidence of AR simulators causing excessive stress to participants. Therefore, AR simulators are a promising tool to be used in medical training, which can provide more emotionally realistic scenarios without the risk of additional harm.

## Introduction

Patient simulators have demonstrated improved learning outcomes in medical training [1-4]. Consequently, over the past decade, the use of simulators has become an increasingly important and prominent part of medical training. These include mechanical manikins (ie, Laerdal SimMan) and the “buddy” system in which a fellow student pretends to be a patient. High-fidelity simulation has been defined as “an opportunity to interact within a realistic clinical environment able to reproduce a wide range of clinical conditions” [5]. The Laerdal SimMan varies by model but is capable of showing respiration, seizures, pupillary changes, auscultatable breath sounds and heart sounds, as well as palpable pulses. However, there is no literature on how realistic these methods are and whether they provoke a realistic emotional response comparable to true emergency medical scenarios in trainees. As such, they may not adequately support the development of critical decision-making behaviors in highly emotional contexts.

To address this lack of realistic emotional context, there has been movement toward using augmented reality (AR) approaches that may substantially improve realism. AR simulation has been increasingly used in medical education over the last decade [6-8]. Most studies regarding AR in medical education focus on the development and initial evaluation of utility and feasibility, particularly in surgical and anatomical education [9-12]. The MedCognition AR system, PerSim, is an augmented reality program used for this study. It uses a HoloLens (Microsoft Corp) headset showing the user a virtual patient who can display various physical exam findings and vitals that are subsequently adjusted by the instructor. Physical exam findings that can be shown include seizures, diaphoresis, retractions, respiratory distress, level of consciousness, and cyanosis, which are not well shown on standard mechanical manikins. HoloLens has been previously shown to be effective in teaching medical students [13].

Problematic here is that it is not known if this increased realism evokes a substantially different stress response in learners than traditional simulation modalities. While a small amount of stress can aid learning outcomes [14], excessive stress could be harmful to the health and well-being of medical trainees. This may be particularly problematic for individuals with certain preexisting psychological traits (eg, psychological disorder and past stressful experiences) that may predispose them to more adverse reactions during training simulation scenarios. There are no existing studies evaluating the psychological or physiological stress response that AR may evoke in learners when used for medical education simulations, and thus, there is a need for systematic evaluation of the educational and safety features of these AR simulations.

There are a variety of ways to assess the physical impact of AR as compared to past manikin approaches. Responses to acute stress, physiologically, are most typically mapped by either the sympathetic response (a general physiological fight-or-flight change that prepares the body for action) or hypothalamic-pituitary-adrenal (HPA) axis activity, which directs a range of hormonal and immune changes in the body [15]. While acute changes are considered adaptive in the face of stress, especially when recovery is swift, at high or prolonged elevated levels, dysfunctions in these systems can lead to health problems. In human studies, HPA axis activity is most typically gauged by salivary cortisol levels, long considered a gold standard marker of acute stress [16]. Similarly, markers of sympathetic activity (eg, galvanic skin responses [GSR]) [17] in response to stressful stimulation have long been considered biomarkers of stress, cognitive load, and attention [18-20].

From the psychological perspective, there are a host of approaches that can assess how AR fares in terms of altering the well-being of those using it. Most obviously, researchers studying acute stressors focus on self-reported measures of acute stress, but also emotional changes such as an increase in negative emotions (eg, fear, anxiety, and sadness) and a decrease in positive emotions (eg, calm and happiness). While some studies have found well-being benefits from the use of certain AR games, the concern is that the negative emotional impact could be severe in medical simulations that depict realistic illness and even death [21,22]. Therefore, we conjectured that assessing both physical and psychological responses to the simulations, as well as less obvious self-report approaches (eg, approaches that detect emotion without overtly asking), is key given the possibility that demand characteristics may alter the ability to identify changes in well-being (eg, medical students may feel uncomfortable admitting feelings of depression or stress, especially in the presence of other students and instructors). This echoes previous calls for multimethod approaches in well-being research [23].

One final important consideration of using emotionally realistic depictions of a traumatic event in AR is the possibility that preexisting psychological experiences may make the simulation more damaging. For example, do individuals coming into a simulation with a history of trauma or depression face potentially aversive psychological or physiological responses, and should these preexisting characteristics be considered risk factors for the use of AR? Past research has not examined this question specifically; however, research has clearly shown that past trauma can be a risk factor for numerous future health and stress concerns [24], and the same can be said for past major stressors and other psychological traits that can similarly predict future disorder [25]. This is thought to be due to individuals with risks such as past traumas resulting in excess stress responses (eg, HPA axis and sympathoadrenal responses), thereby increasing vulnerability to stress-related disease and depression [26-28]. Thus, it is important that with this new approach to teaching, we examine whether certain individuals have excessive stress responses that could be an early indicator of future problems.

In this study, we hypothesized that the higher-fidelity, more realistic AR simulation would more successfully elicit emotional stress compared to a standard manikin simulator. Specifically, we predicted that the AR simulation would be associated with higher levels of negative emotion and self-rated stress, and lower levels of positive emotion as compared to the manikin simulation. Similarly, we hypothesized that the AR simulation would be tied to higher changes in both GSR and salivary cortisol. Finally, we predicted that preexisting psychological traits would not significantly influence the psychological and physiological responses to the simulation.

## Methods

### Participants

The study sample consisted of second-year medical students (N=89) at the University of California, Irvine. All 104 students enrolled in Clinical Foundations II were invited to participate in the study via email. Students were evaluated while completing both AR and standard medical simulation cases on mechanical manikins as part of their training. There were no exclusion criteria, and any medical student who wanted to participate was eligible. The participants were compensated for participating with a $25 Amazon gift card and a free lunch.

### Ethics Approval

This study was approved by both the University of California, Irvine Institutional Review Board (HS#2019-5327, approved October 24, 2019) as well as the US Army Medical Research and Material Command Office of Research Protection (e01201.1a, approved March 18, 2020), and the procedures followed were in accordance with the ethical standards of the responsible committee on human experimentation.

### Study Design

Study sample size (as well as power) was calculated based on a similar previous study and the median salivary cortisol level differences [12]. Using Mann-Whitney *U* test and assuming an alpha of .05 and power of 90%, we calculated a sample size of 44. Allowing for data loss, we planned to enroll 72 learners.

The within-subjects crossover study design allowed for comparison of each student’s psychologic response and minimized confounding due to variance in the individual psychological responses, as students acted as their own controls. The participants were randomized with a random number generator to complete the first case with either the SimMan or PerSim simulation, and subsequently completed the second case with the other modality.

### Procedures

Medical students completed similar medical simulation scenarios, 3 weeks apart, on both a manikin-based simulator, SimMan, and on the AR system, PerSim, while measuring psychological parameters and evidence of stress. Participants had all previously been trained on basic operational procedure for the HoloLens headsets, which provided the hardware for the AR simulation. Before participating in the study sessions, the participants were consented and completed a baseline questionnaire from home, which assessed health behaviors, trait affect, and demographic characteristics relevant to controls. Upon arrival on each study day, the participants were instructed as to what to expect (without disclosing the nature of the simulation), outfitted with an ambulatory wrist or hand GSR monitor and provided a resting salivary cortisol sample. Within each study session, students completed 1 of 2 scenarios centered on pediatric resuscitation and subsequent death of the patient: 1 status asthmaticus and 1 pediatric sepsis, both with unstable vital signs requiring acute resuscitation, who ultimately succumbed to their illness regardless of learner actions. These cases were integrated into the medical student curriculum with the objective of covering personal emotional stressors in work and difficult conversations; however, they also allowed maximum specific psychological effects. Scenarios lasted approximately 10 minutes each.

Electrodermal activity was continuously assessed via wrist monitor before, during, and after the scenario to establish baseline, task (stress reactivity), and recovery periods. Additionally, salivary cortisol samples were collected to align with times before, immediately following, and 15 minutes after each simulation. Psychological data (eg, stress and emotion) were collected through surveys administered before and immediately following each simulation session. The postsimulation survey additionally included qualitative debriefing questions related to the passing of the participant and the medical knowledge of the participant.

### Measures

#### Preexisting Psychological Traits

The preexisting psychological traits that could be considered potential risk factors for adverse reactions were assessed via a survey taken at home before participation in the study. These factors included posttraumatic stress disorder (PTSD), perceived stress, and depression. Posttraumatic stress disorder was assessed with the self-reported 17-item Posttraumatic Stress Disorder-Civilian Checklist, which assesses PTSD symptoms based closely on Diagnostic and Statistical Manual of Mental Disorder, 4th edition criteria [29]. Perceived stress was assessed via the 10-item Perceived Stress Scale [30], which assesses perceptions of stress over the past month. Depression was assessed via the 10-item Center for Epidemiologic Studies Depression Scale Revised, which measures the prevalence of depression symptoms over the past week [31].

#### Self-reported Stress

To measure the perceived stress responses induced from the simulation, slider scales ranging from 1 to 100 were used to capture stress levels before and after the simulation [32]. Participants were asked, “How stressed do you feel right now?” The higher scores indicated more stress.

#### Emotion

##### State Affect

To assess the affective responses to these scenarios, we measured state emotion change (from before to after simulation) using items drawn from the positive and negative affect schedule (PANAS) [33]. Positive and negative affect subscales within the PANAS were used to create variables for positive and negative affect. Mean scores were then calculated for positive and negative affect by using subscales within the PANAS, yielding a positive and negative affect score respectively for each time point.

##### Positive and Negative Word Use

Positive and negative emotion were also assessed via open-answer (qualitative) debriefing surveys following the simulation experiences. These surveys were coded using the Linguistic Inquiry and Word Count program, a validated text analysis software that is widely used in psychological research [34] to count the types of words used in narrative samples. For this study, we used the default positive and negative emotion dictionaries to procure measures tapping the percentage of words of these types in the open responses from participants. This analysis provides an indirect approach to tap the emotional experience of using study simulations.

#### Physiological Stress

##### Salivary Cortisol

Salivary cortisol levels, a known biological correlate of psychological stress [35-37], were monitored throughout the simulations. Samples were collected via the passive drool technique with polypropylene cryovial salivettes at 3 time points that accounted for the lag between biological stress response and hormonal detection in saliva to provide cortisol levels. Timepoints were (1) baseline (before simulation), (2) reactivity (during simulation), and (3) recovery (15 minutes after simulation). Experimental sessions were scheduled between 12 PM and 5 PM to account for the diurnal rhythm of cortisol. Salivettes were stored at –80 °C until batch analysis at the end of data collection at the laboratory of the Institute for Interdisciplinary Salivary Bioscience Research (University of California Irvine, Irvine, CA). Before assaying, the samples were thawed for an hour to return to room temperature. All samples were assayed in duplicate using an expanded-range high-sensitivity salivary cortisol enzyme immunoassay kit (Salimetrics, LLC; State College, PA). The assay range of sensitivity was 0.007 ug/dl to 3.0 ug/dl, and the average intra-assay coefficient of variation was 5.5%.

##### Galvanic Skin Response

The GSR data were collected via a small unobtrusive device (Shimmer3) that was monitored by the researchers throughout the simulations. The device was placed on a wristband that was fastened to participants’ wrists prior to the start of study tasks. To collect GSR data, the device had 2 wires that extended from the hardware and was attached to participants’ palms via 2 electrodes and an additional medical tape when needed to ensure secure connection and a good signal.

Researchers monitored the GSR data using Bluetooth connectivity through a laptop and took notes of any artifacts that could cause spikes in GSR data unrelated to the simulation, such as coughing, external noises, and so on [17]. Additionally, researchers made note of participants who had connectivity issues (eg, due to exceptionally sweaty palms). All these potential artifacts were accounted for during the data cleaning process using an electrodermal activity Analysis application from MindWare Technologies. GSR means were used in the analyses by obtaining the average GSR score for the baseline and reactivity of each simulation session.

#### Simulation Sickness Questionnaire

Adverse side effects were measured with the Simulator Sickness Questionnaire [38,39], a 16-item validated measurement for simulation side effects that have been previously reported in virtual reality literature [40]. This was scored on a scale of 0 to 16 with mean scores calculated and compared with a 2-tailed *t* test.

### Analytic Strategy

Linear mixed model (LMM) for repeated measurements was used for data analysis by using the “MIXED” command in SPSS statistics software (Version 26.0., IBM Corp). Simulation type and time of measurements were considered as fixed effect variables and the participants as random effect variables. A separate LMM analysis was performed for each dependent variable, adjusting for potential confounders accordingly. The correlation between repeated measurements within subjects was considered as “unstructured.” A square root transformation was applied to the Mean GSR and Simulator Sickness Questionnaire, and natural logarithm transformation was applied to cortisol before LMM analysis. A *P* value of less than .05 was considered statistically significant. The changes in outcome measures are presented as mean change (95% CI; *P* value). Similarly, the differences in outcome measures between AR and Manikin simulations are presented as mean (AR-manikin: 95% CI of mean difference; *P* value).

To examine whether perceived stress, depression, and PTSD modify the effect of AR on cortisol and GSR, an LMM analysis was applied to AR data only by including the potential effect modifiers. If the *P* value of a potential effect modifier was greater than .05, its effect modification on the association between AR and dependent variables was excluded.

We first report the psychological impact of the simulations, followed by the physiological impact. Finally, we briefly examine whether there was evidence of moderation due to preexisting psychological traits.

## Results

Of a total of 104 possible participants, 88 (85%) participated. Of these 88 participants, 37 (42%) were male, and 51 (58%) were female medical students with a mean age of 25.2 (SD 2.09, range 22-30) and 24.7 (SD 2.08, range 23-36), respectively.

### Psychological Responses to Simulations

**Figure 1:**
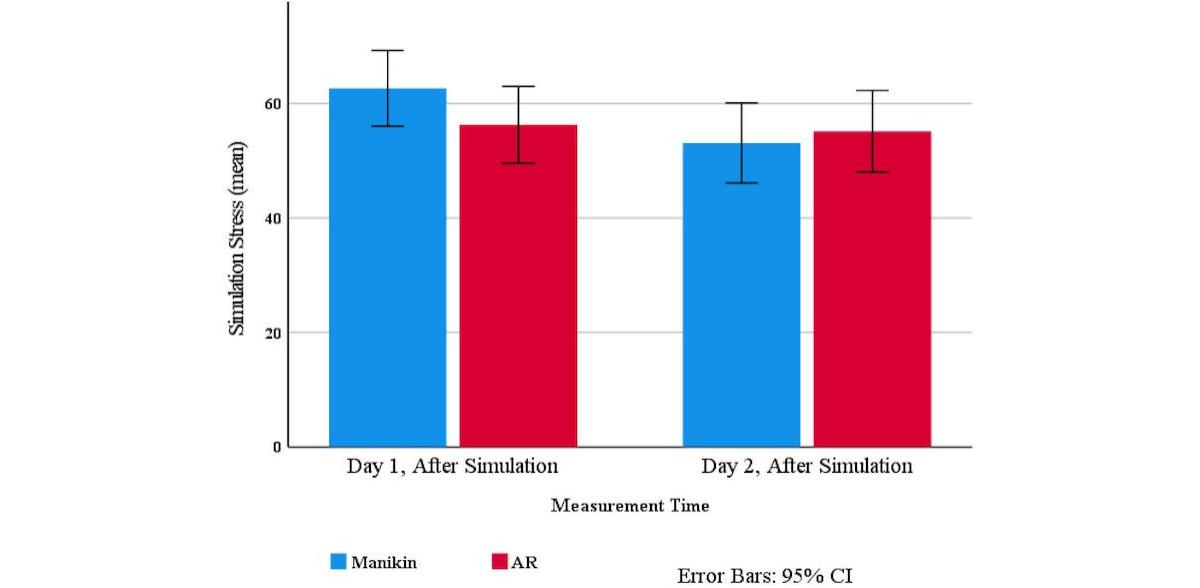
Simulation stress: day 1 vs day 2 (means measured on self-reported 100-point Likert scale). AR: augmented reality.

Negative affect showed an increase of 4.68 (3.57-5.79; *P*<.001) with manikin, and 5.08 (3.96-6.21; *P*<.001) with AR simulation (Table 1). However, the difference between simulation types was not statistically significant, and was adjusted for the day of experiment (AR-manikin: 0.41, 95% CI –0.72 to 1.53; *P*=.48). Similarly, self-reported stress showed an increase of 12.21 (9.53-14.90; *P*<.001) with manikin and 12.75 (10.03-15.47; *P*<.001) with AR simulations (Table 1). However, the difference between simulation types was not statistically significant, and was adjusted for day of experiment and sex of participants (AR-manikin: 0.53, 95% CI –2.35 to 3.42; *P*=.71). Simulation stress (Figure 1) was higher on day 1 compared to day 2 (day 2 minus day 1: –5.29, 95% CI –10.06 to –0.52; *P*=.03; Table 1); however, the difference between the simulation types was not statistically significant and was adjusted for day of experiment and sex of the participants (AR-manikin: –2.17, 95% CI –6.94 to 2.59; *P*=.37). Stress also reached a higher maximum on day 1 (day 2 minus day 1: –6.60, 95% CI –10.49 to –2.72; *P*=.001; Table 1), but this was not related to simulation type after adjusting for day and sex (AR-manikin: –3.02, 95% CI –6.83 to 0.80; *P*=.12). Finally, when examining the open-ended responses to the simulations, there was no statistically significant difference in the percentage of negative emotion word use between simulation types, adjusted for day of experiment, sex, and the word count for Linguistic Inquiry and Word Count (AR-manikin: 0.33, 95% CI –1.12 to 0.46; *P*=.41).

The percentage of positive emotion words used in the narrative descriptions was higher on the first day of simulations (day 2 minus day 1: –0.64, 95% CI –1.18 to –0.10; *P*=.02; Table 1) but there was no statistically significant difference between the simulation types in terms of the percentage of positive emotion words use, which was adjusted for day of experiment, sex of the participants, and total number of words used (AR-manikin: –0.40, 95% CI –0.91 to 0.10; *P*=.12).

**Table 1:** Psychologic responses to simulation.

Measurement and simulation			Measurement time						
			Day 1				Day 2		
			Before simulation		After simulation		Before simulation		After simulation
**Negative affect, mean (SD)**									
	Manikin	14.6 (3.38)		20.5 (5.83)		15.0 (4.35)		18.8 (5.91)	
	AR^a^	13.5 (2.51)		20.7 (6.44)		14.8 (5.20)		18.4 (6.11)	
**Self-reported stress, mean (SD)**									
	Manikin	44.2 (23.24)		57.8 (23.57)		45.1 (19.82)		56.3 (20.14)	
	AR	38.2 (17.87)		54.3 (19.11)		47.5 (27.12)		58.0 (24.72)	
**Simulation stress, mean (SD)**									
	Manikin	N/A^b^		62.6 (21.23)		N/A		53.1 (23.00)	
	AR	N/A		56.3 (22.28)		N/A		55.1 (22.34)	
**Maximum stress, mean (SD)**									
	Manikin	N/A		72.4 (19.19)		N/A		60.0 (24.16)	
	AR	N/A		64.3 (22.22)		N/A		63.0 (23.29)	
**Negative emotion words (%), mean (SD)**									
	Manikin	N/A		6.2 (3.17)		N/A		7.3 (4.10)	
	AR	N/A		6.4 (2.98)		N/A		6.6 (2.67)	
**Positive emotion words (%), mean (SD)**									
	Manikin	N/A		3.0 (1.81)		N/A		2.5 (1.59)	
	AR	N/A		2.7 (1.84)		N/A		2.1 (2.06)	
**Galvanic skin response (√μS), mean (SD)**									
	Manikin	12.9 (8.27)		15.3 (7.67)		11.2 (9.50)		13.6 (7.63)	
	AR	8.8 (6.19)		10.6 (6.11)		12.1 (5.68)		14.1 (4.99)	
**Cortisol (ug/dl), mean (SD)**									
	Manikin	0.2 (0.11)		0.1 (0.09)		0.1 (0.09)		0.1 (0.06)	
	AR	0.1 (0.10)		0.1 (0.13)		0.2 (0.10)		0.2 (0.11)	
**Simulation sickness symptoms score, mean (SD)**									
	Manikin	N/A		23.0 (20.59)		N/A		30.2 (25.78)	
	AR	N/A		27.4 (20.58)		N/A		28.1 (24.68)	

^a^AR: augmented reality.

^b^N/A: not applicable.

### Physiological Responses to Simulation

Manikin and AR simulations were associated with increased GSR (mean change in square root of GSR was 0.38 μS: 0.31-0.46; *P*<.001 and 0.28: 0.20-0.35; *P*<.001, respectively; Table 1). Interestingly, GSR was higher in the manikin group as compared to AR, adjusted for day, sex, and use of any medication by the participants (AR-manikin: –0.11, 95% CI –0.18 to –0.03; *P*=.009).

There was not a statistically significant difference in the mean cortisol level between the simulation groups (Table 1), which was adjusted for the day of experiment, sex of the participants, use of any medication by the participants, and the time past from wakeup to simulation (AR-manikin: 0.04, 95% CI –0.05 to 0.13; *P*=.41). Overall, cortisol was higher in male participants (male minus female: 0.22, 95% CI 0.03-0.40; *P*=.02).

### Simulation Sickness Responses

There was not a statistically significant difference in simulation sickness symptoms’ score between the simulation groups, which was adjusted for day of experiment and sex of the participants (AR-manikin: 0.17, 95% CI –0.29 to 0.62; *P*=.47).

### Moderating Effect of Preexisting Psychological Traits

PTSD (*P*=.39), baseline perceived stress (*P*=.09), and baseline reported depression (*P*=.51) failed to achieve statistical significance when introduced to the model predicting salivary cortisol or GSR based on AR. Thus, we can conclude that these preexisting psychological traits do not predict adverse stress-related outcomes.

## Discussion

### Principal Findings

The goal of this study was to examine whether more realistic AR simulations would be a cause for concern because of potentially high stress, emotion, or physiological responses, especially in a dramatic medical context involving the death of a patient. We did not find a statistically significant difference in the participants’ psychological and physiological reactions to AR and standard medical manikin training simulations. Both the manikin and AR simulators elicited emotional (ie, a reduction in positive emotion and an increase in negative emotion) and elevated stress responses during and after the simulations. However, these psychological responses did not significantly differ between the simulation types.

### Comparison With Prior Work

This finding is consistent with previous studies, which showed that simulation in medical education can elicit a stress response [41-43] as well as a range of emotional and cognitive changes. As these studies suggest, small stress increases are tied to better learning outcomes, which in turn suggests that both modalities of simulation can have a beneficial effect for learners; however, future studies will need to evaluate the actual learning outcomes. Of note, there was some concern that AR might be associated with a dangerously high level of stress because of the added realism and interactive nature; however, it does not seem to be any more stressful than past medical training approaches (ie, manikin here), adding some indication that dangerous levels of stress are not a concern, at least in this simulation. Further subanalysis examining preexisting trauma, perceived stress, and depression did not show statistically significant differences in stress with AR simulation, suggesting that even those with preexisting psychological conditions may not need to be excluded from AR technology in this type of context. Further, stress and negative emotion reported in these simulations do not appear to be at levels that are different compared to other study averages [44-46].

From the physiological stress perspective, this study shows no significant differences between AR and standard manikin simulation technology, except a small difference where the increase in skin conductance in response to the manikin simulator was significantly higher than that of AR—the opposite of what was anticipated. Cortisol differences, however, were not different across the 2 platforms. This suggests that, contrary to expectations, and despite heightened realism and more animated interactions, the AR approach is psychologically and physically comparable to standard manikin-based simulators, and it is perhaps even slightly less physiologically stressful than past learning modalities.

Given the nature of the simulations involving pediatric deaths, it is not surprising that the overall stress increased during and after each simulation. However, students showed decreased stress levels in their second simulation. Previous studies have shown that stress factors in simulation-based training may help with the acquisition of stress management skills [41]. In addition to stress management skills, it could suggest a desensitization to the simulation regardless of type of simulator. Chang et al [47] suggested that VR simulation could be used to desensitize pediatric physicians from stressful situations based on their study evaluating VR stress response and real-life situations. However, Hardernberg et al [48] showed no decreased stress response in nursing students with repeated simulations, which is contradicted with our results. Decreasing levels of stress response could be very useful for educational purposes and future training for many types of medical practitioners who experience high-stress situations.

### Strengths and Limitations

This study is limited as it is a single-site study comparing AR simulation to standard manikin-based simulation. While we attempted to look at multiple evaluators of emotional stress (cortisol, self-reported stress, and electrodermal activity), these still may not have fully captured the stress response of the students. Some students had higher levels of sweat on their palms making GSR data less reliable, as the sensors were more difficult to maintain on their hands. Finally, while we controlled for numerous possible confounders of our biological markers (eg, medication, time of day, and sex), there may be other factors unaccounted for, which may have resulted in bias or noise in the data.

### Conclusions and Implications

AR simulators elicited similar stress responses to manikin-based simulators suggesting they are comparable tools for medical education. Furthermore, there was no evidence of AR simulators causing excessive stress to participants at a level different from existing simulation methods. Future research should evaluate whether AR simulators increase learning outcomes or help with desensitization or stress management skills with repeated use. AR technology is relatively new and its ability to elicit a stress response when compared to standard manikin simulation technology could help guide future educational practices and research.

## Abbreviations

AR: augmented reality
GSR: galvanic skin response
HPA: hypothalamic-pituitary-adrenal
LMM: linear mixed model
PANAS: positive and negative affect schedule
PTSD: posttraumatic stress disorder

## References

[1] Kim J, Park J, Shin S (2016). Effectiveness of simulation-based nursing education depending on fidelity: a meta-analysis. BMC Med Educ.

[2] Pascual JL, Holena DN, Vella MA, Palmieri J, Sicoutris C, Selvan B, Fox AD, Sarani B, Sims C, Williams NN, Schwab CW (2011). Short simulation training improves objective skills in established advanced practitioners managing emergencies on the ward and surgical intensive care unit. J Trauma.

[3] Frost DW, Cavalcanti RB, Toubassi D (2011). Instruction using a high-fidelity cardiopulmonary simulator improves examination skills and resource allocation in family medicine trainees. Simul Healthc.

[4] Maddry JK, Varney SM, Sessions D, Heard K, Thaxton RE, Ganem VJ, Zarzabal LA, Bebarta VS (2014). A comparison of simulation-based education versus lecture-based instruction for toxicology training in emergency medicine residents. J Med Toxicol.

[5] Masotta V, Dante A, Marcotullio A, Bertocchi L, La Cerra C, Caponnetto V, Petrucci C, Alfes CM (2021). The concept of high-fidelity simulation related factors in nursing education: a scoping review. Methodologies and Intelligent Systems for Technology Enhanced Learning, 10th International Conference. Workshops.

[6] Kamphuis C, Barsom E, Schijven M, Christoph N (2014). Augmented reality in medical education?. Perspect Med Educ.

[7] Herron J (2016). Augmented Reality in Medical Education and Training. Journal of Electronic Resources in Medical Libraries.

[8] Tang KS, Cheng DL, Mi E, Greenberg PB (2020). Augmented reality in medical education: a systematic review. Can Med Educ J.

[9] Thomas RG, John NW, Delieu JM (2010). Augmented reality for anatomical education. J Vis Commun Med.

[10] Nicholson DT, Chalk C, Funnell WRJ, Daniel SJ (2006). Can virtual reality improve anatomy education? A randomised controlled study of a computer-generated three-dimensional anatomical ear model. Med Educ.

[11] Zahiri M, Nelson CA, Oleynikov D, Siu K (2018). Evaluation of Augmented Reality Feedback in Surgical Training Environment. Surg Innov.

[12] Shuhaiber JH (2004). Augmented reality in surgery. Arch Surg.

[13] Moro C, Phelps C, Redmond P, Stromberga Z (2020). HoloLens and mobile augmented reality in medical and health science education: A randomised controlled trial. Br. J. Educ. Technol.

[14] Demaria S, Bryson EO, Mooney TJ, Silverstein JH, Reich DL, Bodian C, Levine AI (2010). Adding emotional stressors to training in simulated cardiopulmonary arrest enhances participant performance. Med Educ.

[15] Russell G, Lightman S (2019). The human stress response. Nat Rev Endocrinol.

[16] Dickerson SS, Kemeny ME (2004). Acute stressors and cortisol responses: a theoretical integration and synthesis of laboratory research. Psychol Bull.

[17] Turpin G, Grandfield T (2007). Electrodermal activity. Encyclopedia of Stress.

[18] Johannessen E, Szulewski A, Radulovic N, White M, Braund H, Howes D, Rodenburg D, Davies C (2020). Psychophysiologic measures of cognitive load in physician team leaders during trauma resuscitation. Computers in Human Behavior.

[19] Frith CD, Allen HA (1983). The skin conductance orienting response as an index of attention. Biol Psychol.

[20] Sharma M, Kacker S, Sharma M (2016). A Brief Introduction and Review on Galvanic Skin Response. IJMRP.

[21] Ellis LA, Lee MD, Ijaz K, Smith J, Braithwaite J, Yin K (2020). COVID-19 as 'Game Changer' for the Physical Activity and Mental Well-Being of Augmented Reality Game Players During the Pandemic: Mixed Methods Survey Study. J Med Internet Res.

[22] Harley JM, Lajoie SP, Tressel T, Jarrell A (2020). Fostering positive emotions and history knowledge with location-based augmented reality and tour-guide prompts. Learning and Instruction.

[23] Diener E, Pressman SD, Hunter J, Delgadillo-Chase D (2017). If, Why, and When Subjective Well-Being Influences Health, and Future Needed Research. Appl Psychol Health Well Being.

[24] Westfall NC, Nemeroff CB (2015). The Preeminence of Early Life Trauma as a Risk Factor for Worsened Long-Term Health Outcomes in Women. Curr Psychiatry Rep.

[25] Wild J, Smith KV, Thompson E, Béar F, Lommen MJJ, Ehlers A (2016). A prospective study of pre-trauma risk factors for post-traumatic stress disorder and depression. Psychol Med.

[26] Neigh GN, Gillespie CF, Nemeroff CB (2009). The neurobiological toll of child abuse and neglect. Trauma Violence Abuse.

[27] Starr LR, Hammen C, Conway CC, Raposa E, Brennan PA (2014). Sensitizing effect of early adversity on depressive reactions to later proximal stress: Moderation by polymorphisms in serotonin transporter and corticotropin releasing hormone receptor genes in a 20-year longitudinal study. Dev Psychopathol.

[28] Andersen SL, Tomada A, Vincow ES, Valente E, Polcari A, Teicher MH (2008). Preliminary evidence for sensitive periods in the effect of childhood sexual abuse on regional brain development. J Neuropsychiatry Clin Neurosci.

[29] Wilkins KC, Lang AJ, Norman SB (2011). Synthesis of the psychometric properties of the PTSD checklist (PCL) military, civilian, and specific versions. Depress Anxiety.

[30] Cohen S, Kamarck T, Mermelstein R (1983). A global measure of perceived stress. J Health Soc Behav.

[31] Radloff LS (2016). The CES-D Scale. Applied Psychological Measurement.

[32] Lesage F, Berjot S, Deschamps F (2012). Clinical stress assessment using a visual analogue scale. Occup Med (Lond).

[33] Watson D, Clark LA, Tellegen A (1988). Development and validation of brief measures of positive and negative affect: the PANAS scales. J Pers Soc Psychol.

[34] Tausczik YR, Pennebaker JW (2009). The Psychological Meaning of Words: LIWC and Computerized Text Analysis Methods. Journal of Language and Social Psychology.

[35] Takai N, Yamaguchi M, Aragaki T, Eto K, Uchihashi K, Nishikawa Y (2004). Effect of psychological stress on the salivary cortisol and amylase levels in healthy young adults. Arch Oral Biol.

[36] Alberdi A, Aztiria A, Basarab A (2016). Towards an automatic early stress recognition system for office environments based on multimodal measurements: A review. J Biomed Inform.

[37] van Eck M, Berkhof H, Nicolson N, Sulon J (1996). The effects of perceived stress, traits, mood states, and stressful daily events on salivary cortisol. Psychosom Med.

[38] Sequeira H, Hot P, Silvert L, Delplanque S (2009). Electrical autonomic correlates of emotion. Int J Psychophysiol.

[39] Bouchard S, Robillard G, Renaud P (2007). Revising the factor structure of the Simulator Sickness Questionnaire. Annual Review of CyberTherapy and Telemedicine.

[40] Moro C, Štromberga Z, Raikos A, Stirling A (2017). The effectiveness of virtual and augmented reality in health sciences and medical anatomy. Anat Sci Educ.

[41] Andreatta PB, Hillard M, Krain LP (2010). The impact of stress factors in simulation-based laparoscopic training. Surgery.

[42] Dias RD, Scalabrini Neto A (2017). Acute stress in residents during emergency care: a study of personal and situational factors. Stress.

[43] Keitel A, Ringleb M, Schwartges I, Weik U, Picker O, Stockhorst U, Deinzer R (2011). Endocrine and psychological stress responses in a simulated emergency situation. Psychoneuroendocrinology.

[44] Cohen S, Janicki-Deverts D (2012). Who's stressed? Distributions of psychological stress in the United States in probability samples from 1983, 2006 and 2009. J Appl Soc Psychol.

[45] Cohen S (1988). Perceived stress in a probability sample of the United States. The Social Psychology of Health.

[46] Warttig SL, Forshaw MJ, South J, White AK (2013). New, normative, English-sample data for the Short Form Perceived Stress Scale (PSS-4). J Health Psychol.

[47] Chang TP, Beshay Y, Hollinger T, Sherman JM (2019). Comparisons of Stress Physiology of Providers in Real-Life Resuscitations and Virtual Reality-Simulated Resuscitations. Simul Healthc.

[48] Hardenberg J, Rana I, Tori K (2020). Evaluating Impact of Repeated Exposure to High Fidelity Simulation: Skills Acquisition and Stress Levels in Postgraduate Critical Care Nursing Students. Clinical Simulation in Nursing.

[49] The U.S. Army Medical Research and Development Command.

[50] Medical Technology Enterprise Consortium.

